# A Dual‐Membrane Biomimetic Nanoplatform Enables Triple‐Modal Therapy Against SARS‐CoV‐2 Through Viral Decoy, Inflammation Neutralizing, and Intracellular RNAi

**DOI:** 10.1002/advs.77581

**Published:** 2026-09-06

**Authors:** Hui Li, Bohan Zhang, Chao Shang, Yi Li, Te Zhao, Shengya Liu, Dan Liu, Junwei Che, Cuiling Zhang, Pinghui Wu, Wenjie Yang, Junjie Ding, Yuhua Ran, Yanan Zhai, Haihua Xiao, Jing Gao, Xiao Li, Chunsheng Gao, Lin Li, Zhiping Li

**Affiliations:** ^1^ State Key Laboratory of National Security Specially Needed Medicines Beijing China; ^2^ Academy of Military Medical Sciences Beijing China; ^3^ Department of Pharmacy Peking University Third Hospital Beijing China; ^4^ State Key Laboratory of Pathogen and Biosecurity Beijing China; ^5^ Changchun Veterinary Research Institute Chinese Academy of Agricultural Sciences Changchun China; ^6^ Shenzhen International Travel Health Care Center (Shenzhen Customs District Port Outpatient Clinics) Shenzhen China; ^7^ Institute of Chemistry Chinese Academy of Sciences Beijing China

**Keywords:** biomimetic nanoplatform, COVID‐19, cytokine storm neutralization, RNA interference, triple‐modal therapy, viral decoy

## Abstract

The persistent evolution of SARS‐CoV‐2 and the concomitant risk of life‐threatening hyperinflammation, such as cytokine storm syndrome, underscore the urgent need for therapeutic strategies that simultaneously target viral replication and dysregulated host immunity. Herein, we describe a dual‐membrane biomimetic nanoplatform, designated [A&T]MLN, comprising siRNA‐loaded lipid nanoparticles with a hybrid membrane derived from ACE2‐overexpressing HEK293T cells and THP‐1 macrophages. This design integrates a high‐density viral decoy based on ACE2 with the inherent immunomodulatory capacity of macrophage membranes. [A&T]MLN demonstrates broad‐spectrum and potent neutralization of diverse SARS‐CoV‐2 variants by competitively blocking viral entry, while actively scavenging key inflammatory cytokines (including IL‐6, IL‐1β, and TNF‐α) via membrane‐displayed receptors. In a murine model of acute lung injury that recapitulates COVID‐19 immunopathology, [A&T]MLN treatment significantly attenuated pulmonary inflammation and tissue damage. Additionally, the platform enabled efficient cytosolic delivery of siRNA, establishing a third modality for intracellular suppression of viral gene expression. Collectively, [A&T]MLN represents a complementary triple‐modal therapy that simultaneously addresses viral entry, intracellular replication, and hyperinflammation (the core interconnected pathologies of severe COVID‐19), offering a versatile and adaptive strategy against evolving SARS‐CoV‐2 variants and related inflammatory syndromes.

## Graphical abstract

[A&T]MLN is a triple‐modal nanoplatform with siRNA‐loaded LN coated with hybrid ACE2/macrophage membrane. It blocks viral entry, neutralizes IL‐6/IL‐1β/TNF‐α, and delivers siRNA to suppress viral replication. In a murine lung injury model, it attenuates inflammation, offering a multi‐pronged strategy against SARS‐CoV‐2 variants and hyperinflammation.

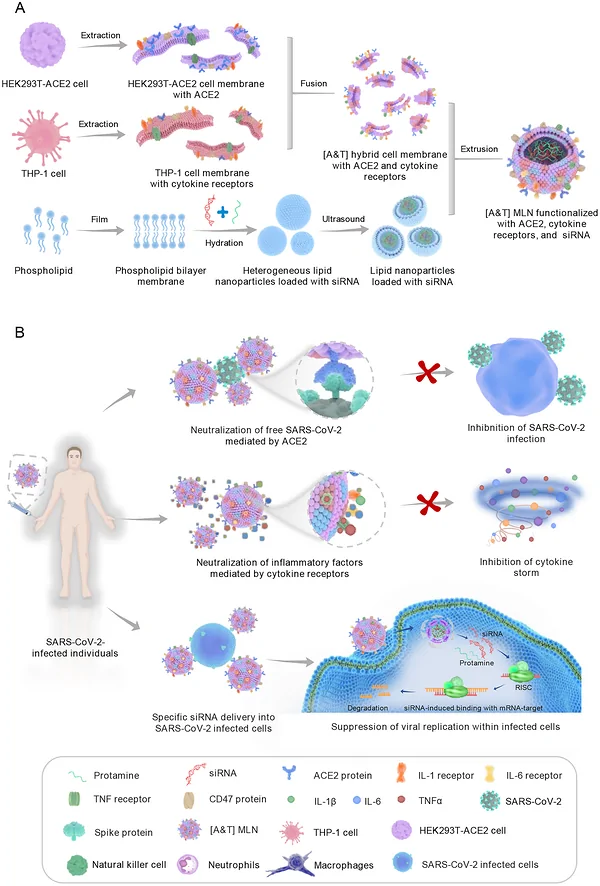

## 1 Introduction

The ongoing Coronavirus Disease 2019 (COVID‐19) pandemic, caused by the severe acute respiratory syndrome coronavirus 2 (SARS‐CoV‐2), remains a persistent threat to global health [1, 2, 3]. Continued viral evolution, the burden of long‐term sequelae, and the limitations of current therapies collectively highlight the urgent need for more comprehensive treatment strategies [4, 5, 6, 7, 8]. Although vaccines reduce severe outcomes, therapeutic interventions for established infection remain critical, particularly in immunocompromised and high‐risk populations [9, 10, 11].

Current clinical management largely separates antiviral and anti‐inflammatory approaches [12, 13, 14, 15]. Direct antivirals, such as monoclonal antibodies and protease inhibitors, aim to reduce viral load but are hampered by rapid viral mutation and limited intracellular efficacy [16, 17]. Immunomodulators like corticosteroids temper hyperinflammation but may suppress beneficial immune responses and increase the risk of secondary infections [18, 19, 20]. This fragmented strategy fails to address the intertwined viral‐host pathology in a coordinated manner, thereby highlighting a clear therapeutic unmet need.

An ideal therapy would simultaneously block viral entry, suppress intracellular replication, and mitigate pathological inflammation, thereby acting at multiple stages of the infection cascade. Such a multimodal strategy could enhance efficacy, lower effective doses, reduce resistance development, and improve clinical outcomes by disrupting the virus‐host feedback loop in a complementary manner.

**FIGURE 1:**
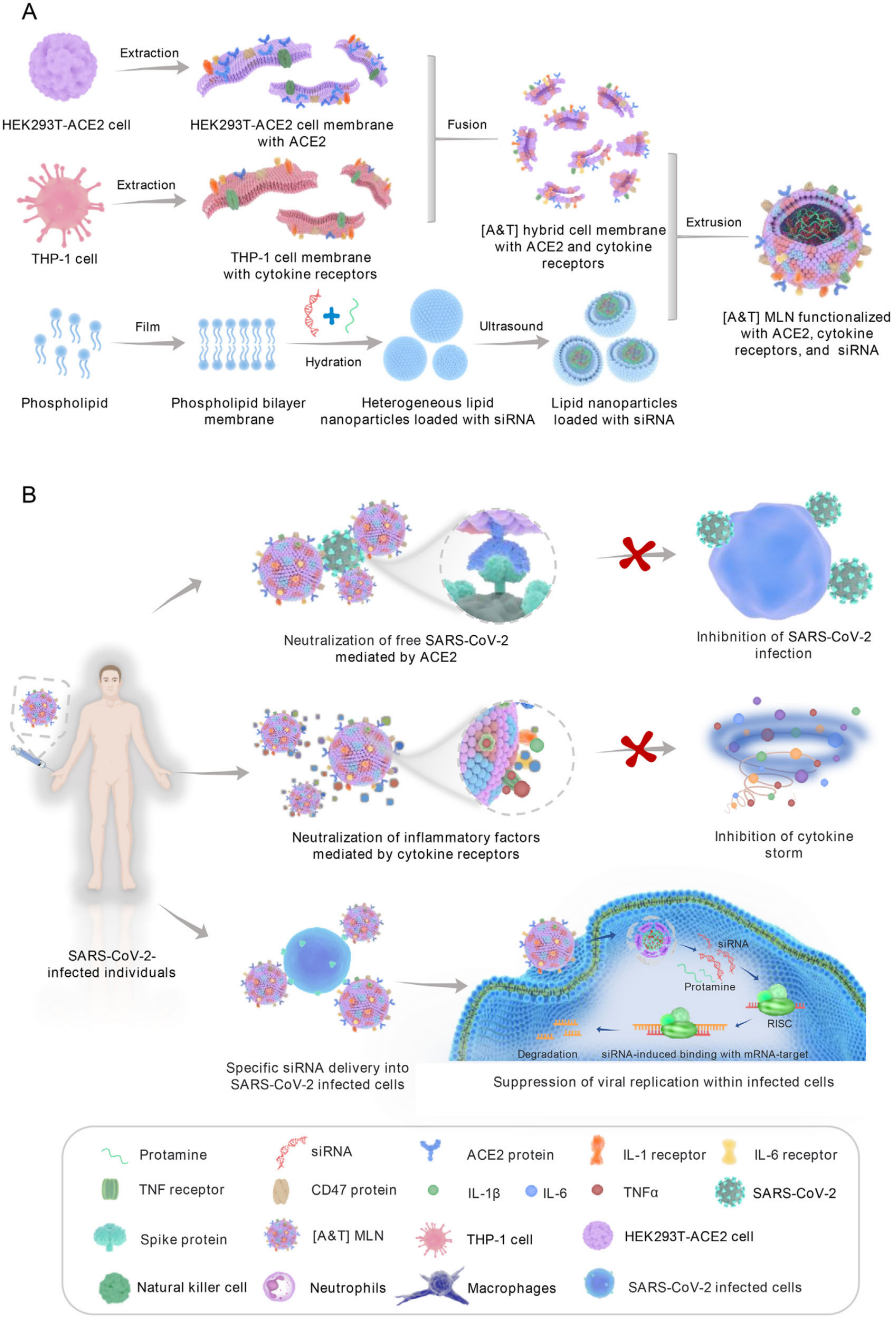
Schematic design and proposed therapeutic mechanism of the dual‐membrane biomimetic nanoplatform [A&T]MLN. (A) Fabrication process of [A&T]MLN. Cell membranes isolated from ACE2‐overexpressing HEK293T cells and THP‐1‐derived macrophages are fused and subsequently coated onto siRNA‐loaded lipid nanoparticles (prepared by thin‐film hydration) to generate the final nanoconstruct. (B) Proposed triple‐modal therapeutic action against SARS‐CoV‐2 infection: (i) neutralization of free virions via high‐affinity binding of surface‐displayed ACE2; (ii) attenuation of the cytokine storm through scavenging of key inflammatory mediators (e.g., IL‐6, TNF‐α) by macrophage‐derived membrane receptors; and (iii) intracellular delivery of therapeutic siRNA to suppress viral replication.

In this study, we describe a biomimetic dual‐membrane nanoplatform that co‐delivers three therapeutic actions for complementary anti‐SARS‐CoV‐2 benefit (Figure 1A). The core construct consists of a lipid nanoparticle coated with fused cell membranes derived from two sources: (1) ACE2‐overexpressing cells, which provide viral decoy receptors to neutralize circulating virions and prevent cellular entry; and (2) macrophages, which enable inflammatory tropism and homing to infected lung tissues for localized cytokine neutralization. Within the nanoparticles, we further encapsulated small‐interfering RNA (siRNA) designed to silence conserved viral genes that are critical for intracellular replication.

This triple‐modal nanoplatform is designed to operate in a sequential manner (Figure 1B): first, the ACE2‐enriched membrane serves as a decoy to capture and neutralize free virus particles; second, the macrophage membrane promotes targeted accumulation at inflammatory sites and directly neutralizes pro‐inflammatory cytokines; and third, upon cellular internalization, the released siRNA silences viral gene expression, thereby effectively inhibiting intracellular viral replication. We hypothesize that this coordinated antiviral, anti‐inflammatory, and RNAi strategy will provide more complete and durable suppression of infection and associated immunopathology. In this study, we detail the design, synthesis, and mechanistic validation of this integrated nanoplatform, demonstrate its complementary efficacy both in vitro and in vivo, and propose it as a next‐generation therapeutic candidate against COVID‐19 and related emerging viral diseases.

## 2 Results

### 2.1 Engineering Functional Hybrid Membranes for Nanoplatform Construction

**FIGURE 2:**
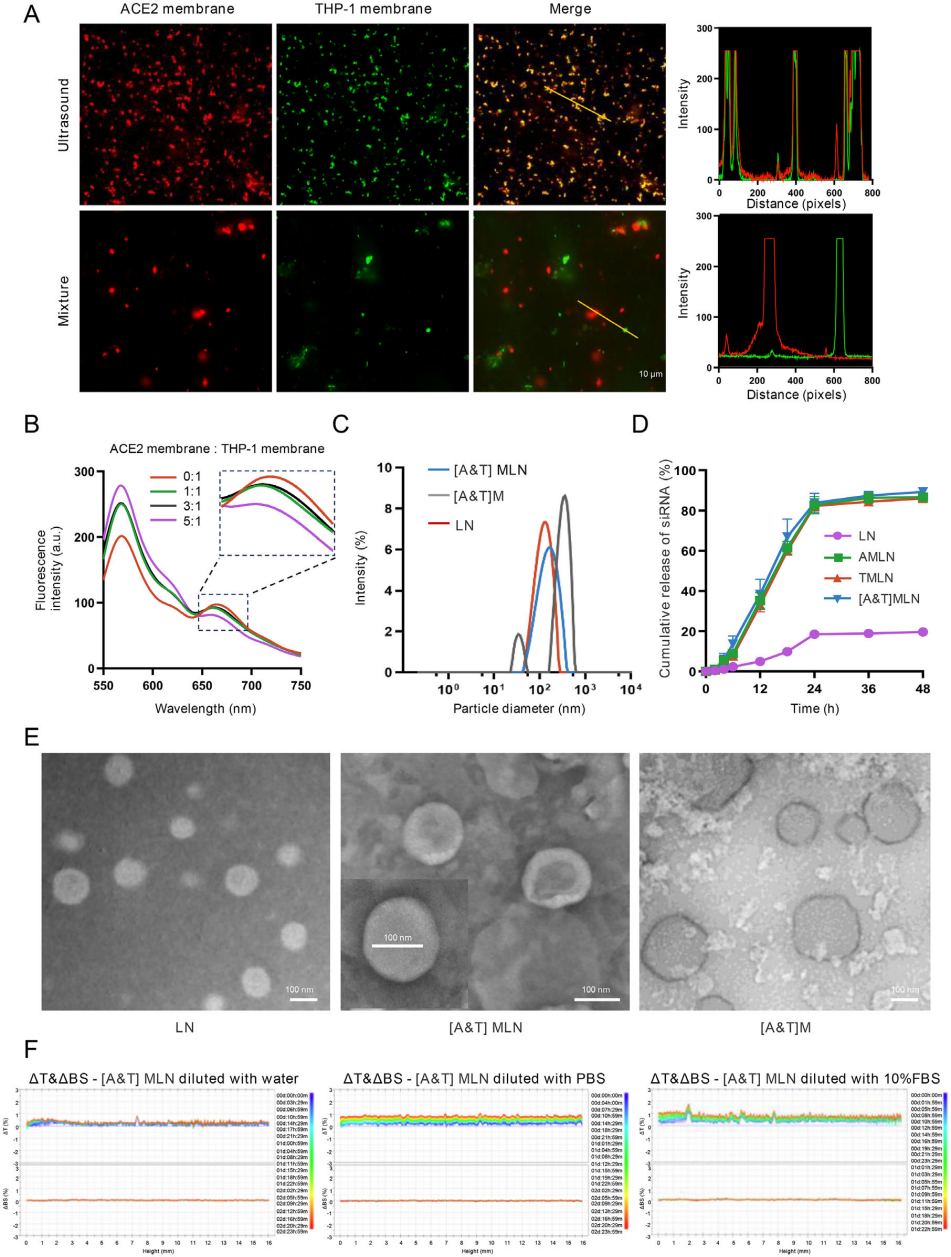
Fabrication, characterization, and biophysical properties of [A&T]MLN. (A) Validation of membrane fusion. Confocal microscopy images of fused hybrid membranes vs. a physical mixture of DiI‐labeled ACE2 membranes (red) and DiO‐labeled macrophage membranes (green). Fluorescence intensity profiles along the yellow lines in the overlay images are shown below. Scale bar, 10 µm. (B) Quantification of membrane fusion by Förster resonance energy transfer (FRET). Donor (DiI) fluorescence recovery after mixing labeled macrophage membranes with ACE2 membranes at the indicated weight ratios confirms efficient membrane merging. (C) Hydrodynamic size distribution of [A&T]MLN, hybrid membrane vesicles ([A&T]M), and lipid nanoparticles (LN), measured by dynamic light scattering. (D) siRNA release profiles. Cumulative in vitro release of Cy5‐siRNA from [A&T]MLN in PBS (pH 7.4) at 37°C. Data are presented as mean ± SD (*n* = 3). (E) Morphology. Representative transmission electron microscopy (TEM) images of negatively stained bare LN, [A&T]MLN, and [A&T]M. Scale bar, 100 nm. (F) Colloidal stability. Transmission (ΔT) and backscattering (ΔBS) variations of [A&T]MLN over 72 h in DEPC‐water, PBS (pH 7.4), and complete culture medium (RPMI‐1640 supplemented with 10% FBS), measured by Turbiscan analysis at 37°C.

Prior to nanoparticle assembly, plasma membranes were isolated from HEK293T‐ACE2 and THP‐1 cells via differential centrifugation. Confocal microscopy of pre‐stained cells confirmed the high purity of the membrane extracts, with no detectable nuclear contamination (Figure S1A–C). Western blot (WB) and enzyme‐linked immunosorbent assay (ELISA) analyses confirmed robust ACE2 overexpression in the HEK293T‐ACE2 membranes, exhibiting an approximately 16‐fold higher concentration than that observed in native HEK293T membranes (Figure S2A,B), with consistent expression across multiple production batches. Successful fusion of the two membrane types was further validated by colocalization imaging, which demonstrated uniform yellow fluorescence in the fused preparation, in contrast to the distinct red and green signals observed in a physical mixture (Figure 2A). Förster resonance energy transfer (FRET) analysis corroborated efficient membrane merging, as indicated by donor fluorescence (DiI, 565 nm) recovery (Figure 2B).

### 2.2 Synthesis and Characterization of the [A&T]MLN Nanoplatform

The dual‐membrane‐coated nanoplatform ([A&T]MLN) was fabricated and comprehensively characterized. Transmission electron microscopy (TEM) confirmed its spherical core–shell morphology (Figure 2E). Dynamic light scattering measurements revealed hydrodynamic diameters of 149.1 ± 7.8 nm for [A&T]MLN and 313.9 ± 16.1 nm for the hybrid membrane vesicles ([A&T]M) (Figure 2C). The zeta potential shifted from +18.6 mV for bare LN and −20.5 mV for [A&T]M to +7.4 mV for [A&T]MLN. The siRNA encapsulation efficiency reached 93.3 ± 1.1%. In vitro release studies demonstrated that membrane‐coated nanoparticles (including [A&T]MLN) achieved >80% siRNA release within 24 h, significantly higher than that observed for bare LN (∼20%) (Figure 2D). Moreover, [A&T]MLN exhibited excellent colloidal stability in various biological media, with transmission and backscattering variations remaining below 3% over 72 h (Figure 2F).

### 2.3 Molecular Basis for Virus Binding and Cytokine Neutralization

**FIGURE 3:**
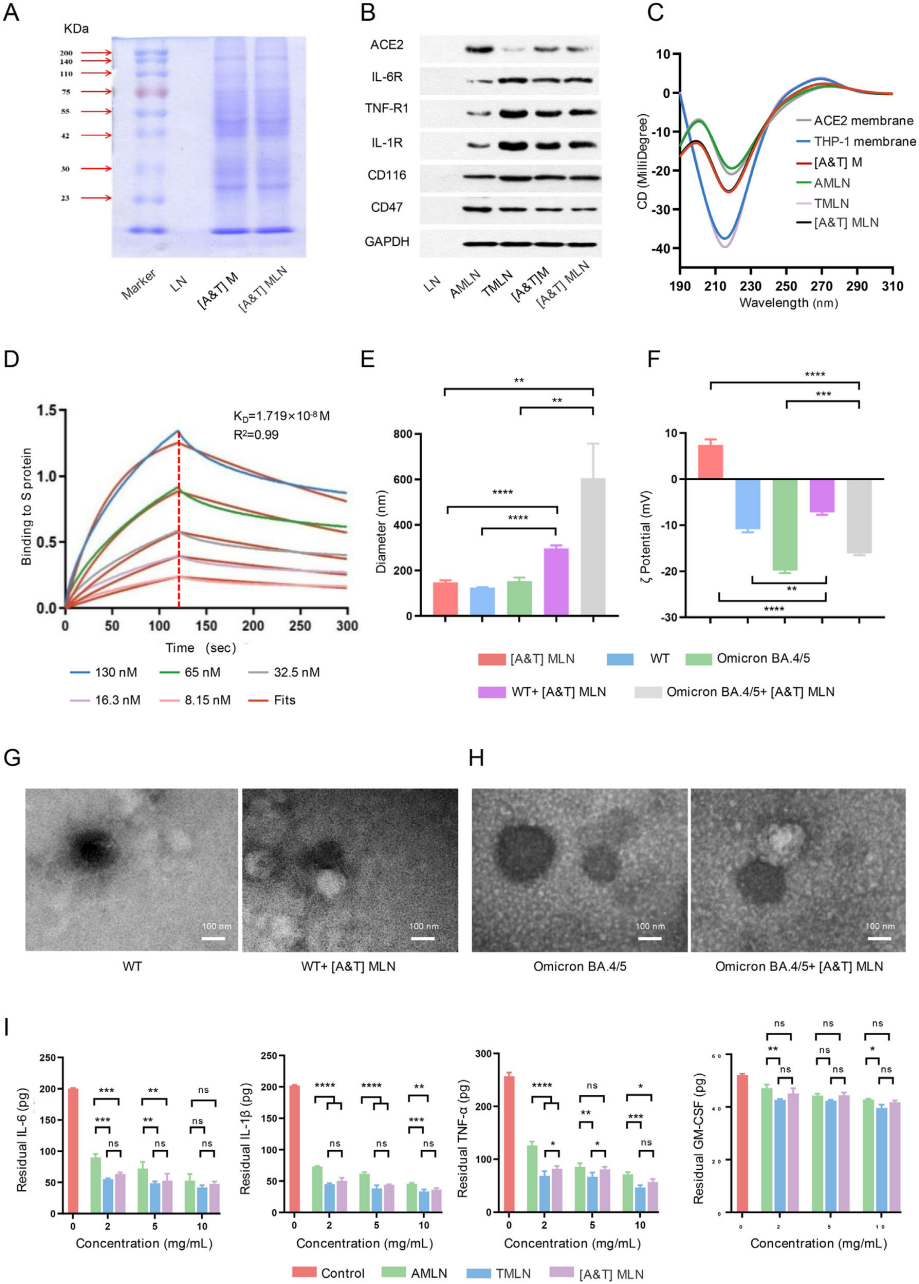
Molecular basis for viral interaction and cytokine neutralization. (A) Protein composition. Coomassie blue‐stained SDS‐PAGE gel of proteins extracted from [A&T]MLN and [A&T]M; LN serves as a negative control. (B) Presence of key functional proteins. Western blot analysis confirming the transfer of ACE2, IL‐6R, TNF‐R1, IL‐1R, CD116, and CD47 to [A&T]MLN and [A&T]M; LN serves as a negative control. (C) Protein secondary structure integrity. Far‐UV circular dichroism (CD) spectra of proteins extracted from [A&T]MLN, THP‐1 membrane, [A&T]M, AMLN, and TMLN. (D) Binding kinetics to SARS‐CoV‐2 spike protein. Biolayer interferometry (BLI) sensorgrams of serially diluted [A&T]MLN (62.5–1000 nm) binding to immobilized spike S1+S2 ECD. Red lines represent the global fit to a 1:1 binding model (*R* ^2^ = 0.99). (E, F) Virus‐induced changes in nanoparticle properties. Hydrodynamic diameter (E) and zeta potential (F) of [A&T]MLN after 2 h incubation with authentic wild‐type (WT) or Omicron BA.5 SARS‐CoV‐2. Data are presented as mean ± SD (*n* = 3). Statistical significance was determined by one‐way ANOVA. ns, not significant; ^**^ *P* < 0.01; ^***^ *P* < 0.001; ^****^ *P* < 0.0001. (G, H) Visualization of virus‐nanoparticle complexes. Representative TEM images of [A&T]MLN bound to authentic SARS‐CoV‐2 WT (G) or Omicron BA.5 (H). Scale bars, 100 nm. (I) In vitro cytokine neutralization. Residual levels of human recombinant IL‐6, IL‐1β, TNF‐α, and GM‐CSF after incubation with AMLN, TMLN, or [A&T]MLN (0–10 µg/mL), quantified by ELISA. Data are presented as mean ± SD (*n* = 3). Statistical significance was determined by one‐way ANOVA. ns, not significant; ^*^ *P* < 0.05; ^**^ *P* < 0.01; ^***^ *P* < 0.001; ^****^ *P* < 0.0001.

Western blot and SDS‐PAGE analyses confirmed the successful transfer of key functional proteins (including ACE2, IL‐6R, IL‐1R, TNF‐R1, CD116 and CD47) from the source membranes to [A&T]MLN (Figure 3A,B). Circular dichroism spectroscopy indicated that protein secondary structure was preserved during fabrication (Figure 3C). The interaction between [A&T]MLN and the SARS‐CoV‐2 spike protein was quantified using biolayer interferometry (BLI). Analysis of the binding kinetics yielded an equilibrium dissociation constant (K_D_) of 1.72 × 10^−8^ m (Figure 3D), confirming high‐affinity binding mediated by the surface‐displayed ACE2. Incubation of [A&T]MLN with authentic wild‐type or Omicron BA.5 virions resulted in a significant increase in hydrodynamic diameter (Figure 3E) and a shift in zeta potential (Figure 3F), consistent with complex formation. TEM further visualized the adhesion of [A&T]MLN to both viral variants (Figure 3G,H). The cytokine‐neutralizing capacity was assessed in vitro by incubating [A&T]MLN with recombinant human IL‐6, IL‐1β, TNF‐α, and GM‐CSF. ELISA revealed a dose‐dependent reduction in all cytokines tested (Figure 3I). [A&T]MLN and TMLN exhibited superior clearance of IL‐6, IL‐1β and TNF‐α compared to AMLN. At 10 µg, [A&T]MLN neutralized 76.2 ± 1.9% of IL‐6, 82.1 ± 1.2% of IL‐1β, 77.7 ± 2.1% of TNF‐α, and 19.6 ± 1.2% of GM‐CSF.

### 2.4 Potent and Broad‐Spectrum Antiviral Activity Against SARS‐CoV‐2

**FIGURE 4:**
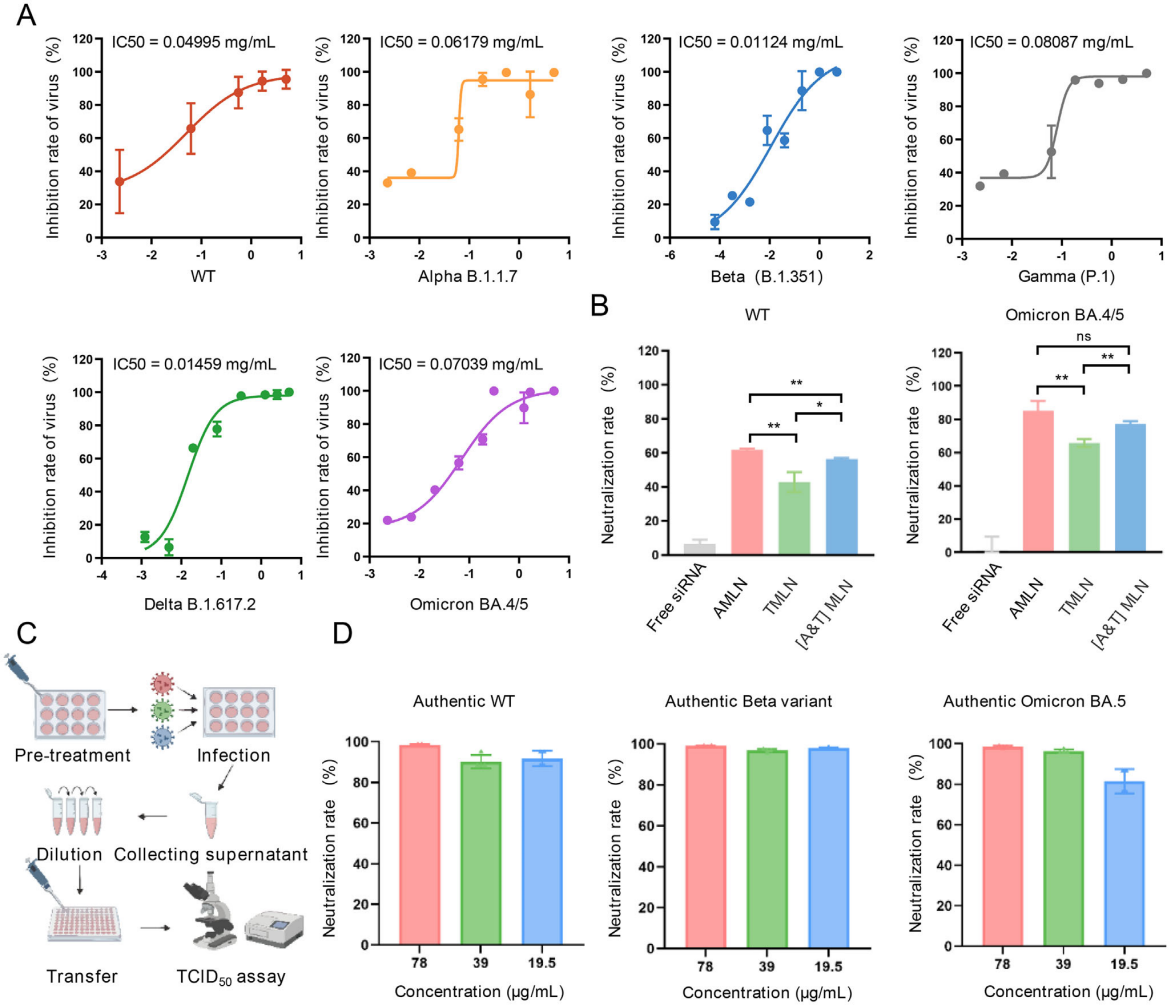
Broad‐spectrum antiviral activity of [A&T]MLN against SARS‐CoV‐2. (A) Dose‐response neutralization of pseudotyped SARS‐CoV‐2 variants. Neutralization activity of [A&T]MLN against a panel of pseudoviruses (WT, Alpha, Beta, Gamma, Delta, Omicron BA.4/5) was measured by a luciferase‐reporter assay. Data are presented as mean ± SD (*n* = 3). (B) Contribution of the ACE2 membrane to neutralization potency. Neutralization of WT and Omicron BA.4/5 pseudoviruses by [A&T]MLN was compared to that of nanoparticles lacking the ACE2 membrane. Data are presented as mean ± SD (*n* = 3). Statistical significance was determined by one‐way ANOVA. ns, not significant; ^*^ *P* < 0.05; ^****^ *P* < 0.0001. (C) Schematic illustration of the authentic virus neutralization assay. (D) Neutralization of authentic SARS‐CoV‐2 variants. Vero E6 cells pretreated with [A&T]MLN (19.5–78 µg/mL) were infected with WT, Beta, or Omicron BA.5 viruses (MOI = 0.01). Viral titers were determined by TCID_50_. Data are presented as mean ± SD (*n* = 3).

The neutralization breadth of [A&T]MLN was assessed against a panel of six pseudotyped SARS‐CoV‐2 variants. [A&T]MLN neutralized all variants with a geometric mean half‐maximal inhibitory concentration (IC50) of 48.1 µg/mL, demonstrating 100% breadth (Figure 4A). In a direct comparison using WT and Omicron BA.4/5 pseudoviruses, both [A&T]MLN and AMLN exhibited significantly stronger neutralization than TMLN (Figure 4B). The efficacy against authentic viruses was further validated, with [A&T]MLN potently inhibiting infection by WT, Beta, and Omicron BA.5 variants in a dose‐dependent manner, reducing viral titers by up to 3 log units and achieving inhibition rates of > 98% at the highest concentration (Figure 4D). Collectively, these data establish [A&T]MLN as a potent and broad‐spectrum antiviral agent capable of high‐affinity binding and functional neutralization of diverse SARS‐CoV‐2 variants.

### 2.5 Effective Attenuation of Cytokine Storm and Lung Injury In Vivo

**FIGURE 5:**
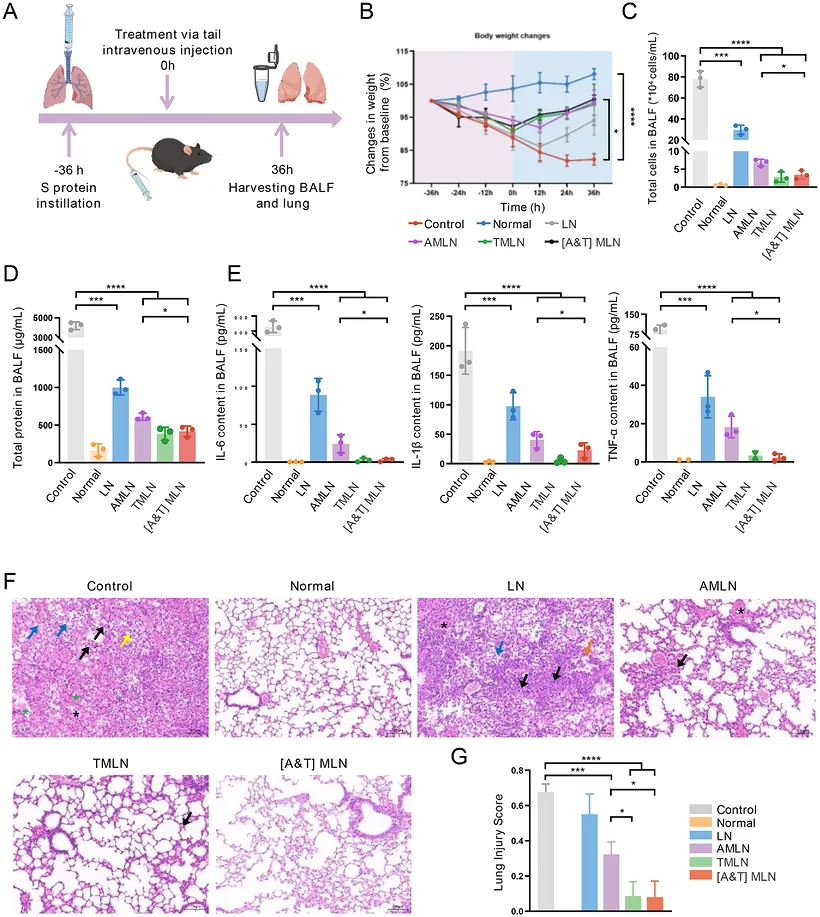
In vivo attenuation of cytokine storm and acute lung injury. (A) Schematic illustration of the acute lung injury (ALI) model and treatment timeline. (B) Body weight changes of K18‐hACE2 mice post‐treatment. (C) Total cell counts and (D) total protein concentration in bronchoalveolar lavage fluid (BALF). (E) Levels of inflammatory cytokines (IL‐6, IL‐1β, TNF‐α) in BALF. Data in (B–E) are presented as mean ± SD (*n* = 5). Statistical significance was determined by one‐way ANOVA. ^*^ *P* < 0.05; ^***^ *P* < 0.001; ^****^ *P* < 0.0001. (F) Representative H&E‐stained lung sections at 48 h post‐treatment. Pathological features: black arrows, neutrophil infiltration; yellow arrows, macrophages; blue arrows, lymphocytes; orange arrows, disrupted bronchial structure; asterisks, red blood cells (black) or proteinaceous debris (green). Scale bar, 100 µm. (G) Quantitative lung injury scores. Data are presented as mean ± SD (*n* = 5). Statistical significance was determined by one‐way ANOVA. ^*^ *P* < 0.05; ^***^ *P* < 0.001; ^****^ *P* < 0.0001.

The in vivo anti‐inflammatory efficacy was evaluated in K18‐hACE2 mice with acute lung injury induced by the SARS‐CoV‐2 spike S1 subunit protein (S1SP) (Figure 5A). Nanoparticles were administered at 36 h post‐induction, which reversed S1SP‐induced weight loss (Figure 5B) and significantly attenuated elevations in bronchoalveolar lavage fluid (BALF) total cell counts, total protein levels, and inflammatory cytokines (IL‐6, IL‐1β, and TNF‐α), with [A&T]MLN and TMLN being the most effective (Figure 5C–E). Histopathological analysis (Figure 5F) and quantitative lung injury scores (LIS) (Figure 5G) confirmed the therapeutic superiority of membrane‐coated nanoparticles. While bare LN showed only minor improvement (LIS = 0.55 ± 0.12) and AMLN provided moderate protection (LIS = 0.29 ± 0.06), [A&T]MLN and TMLN restored lung architecture to near‐normal states, with LIS values of 0.08 ± 0.09 and 0.09 ± 0.08, respectively (both* P* < 0.0001 vs. control; *P* < 0.05 vs. AMLN).

### 2.6 Efficient Macrophage Evasion and Cytosolic siRNA Delivery

**FIGURE 6:**
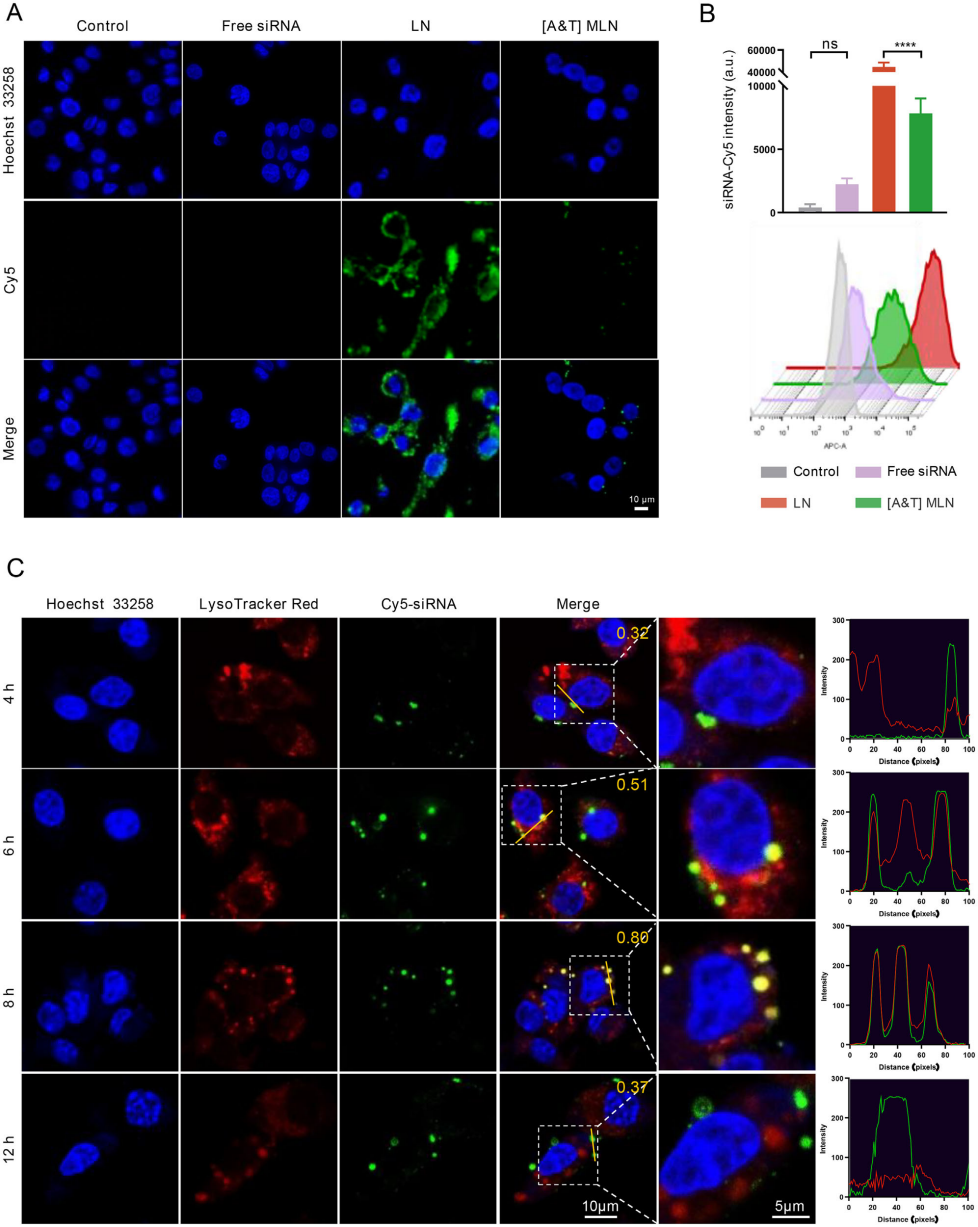
Cellular evasion, uptake, and intracellular trafficking of siRNA. (A) Qualitative cellular uptake. Confocal laser scanning microscopy (CLSM) images of THP‐1‐derived macrophages incubated for 8 h with Cy5‐siRNA‐loaded [A&T]MLN or LN (300 nm siRNA). Nuclei were stained with Hoechst 33258 (blue). Scale bar, 10 µm. (B) Quantitative cellular uptake. Flow cytometric analysis of Cy5 fluorescence intensity in macrophages treated as in (A). Data are presented as mean ± SD (*n* = 3). Statistical significance was determined by one‐way ANOVA. *ns*, not significant; ^****^ *P* < 0.0001. (C) Time‐dependent intracellular trafficking and lysosomal escape. CLSM images of RAW 264.7 cells treated with Cy5‐siRNA‐loaded [A&T]MLN (green). Lysosomes/endosomes were stained with LysoTracker Red (red); nuclei were stained with Hoechst (blue). Insets show magnified views. Fluorescence intensity profiles along the yellow lines and the corresponding Pearson's correlation coefficients (*R*) are shown. Scale bars: overview, 10 µm; insets, 5 µm.

The ability of membrane‐coated nanoparticles to evade phagocytic clearance was assessed using PMA‐differentiated THP‐1 macrophages. Confocal laser scanning microscopy (CLSM) revealed significantly lower cellular uptake of [A&T]MLN compared to uncoated LN (Figure 6A), a finding further corroborated by quantitative flow cytometry (Figure 6B). The intracellular fate of siRNA delivered by [A&T]MLN was tracked over time using Cy5‐labeled siRNA (Figure 6C). At 4 h post‐treatment, siRNA and lysosomes showed minimal colocalization (Pearson's *R* = 0.32). Colocalization increased by 6 h (*R* = 0.51), indicating endosomal entrapment, and peaked at 8 h (*R* = 0.80). By 12 h, the fluorescent signal became dispersed (*R* = 0.37), with siRNA fluorescence diffusing into the cytosol, thereby demonstrating successful endosomal escape and cytoplasmic release.

### 2.7 Prolonged Systemic Circulation and Favorable Biodistribution

**FIGURE 7:**
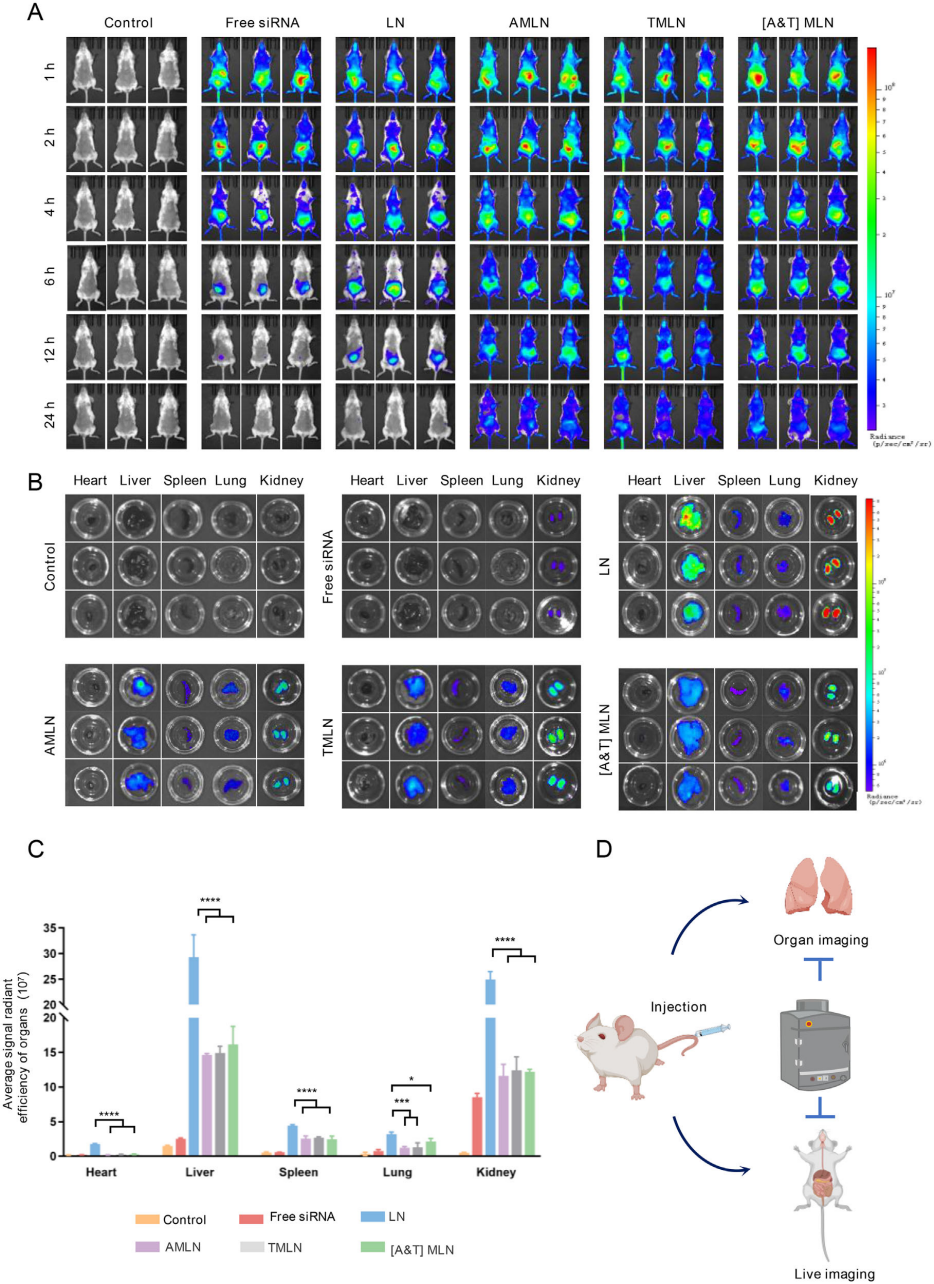
Pharmacokinetics and biodistribution profile. (A) In vivo whole‐body fluorescence imaging at indicated time points after intravenous injection of PBS, free Cy5‐siRNA, or Cy5‐siRNA‐loaded nanoparticles (LN, AMLN, TMLN, [A&T]MLN; siRNA dose: 1.2 mg/kg). (B) Ex vivo organ distribution. Representative fluorescence images of major organs harvested 4 h post‐injection from mice in (A). (C) Quantitative analysis of fluorescence intensity in organs from (B). Data are presented as mean ± SD (*n* = 3 per group). Statistical significance was determined by one‐way ANOVA. ^*^ *P* < 0.05; ^***^ *P* < 0.001; ^****^ *P* < 0.0001. (D) Schematic illustration of the biodistribution study design.

The circulation kinetics of [A&T]MLN were assessed by real‐time in vivo imaging in peripheral blood mononuclear cell (PBMC)‐humanized mice (Figure 7A). Free Cy5‐siRNA and LN‐encapsulated Cy5‐siRNA were rapidly cleared from the bloodstream, largely disappearing from circulation within 6 and 12 h, respectively. In contrast, membrane‐coated nanoparticles (AMLN, TMLN, and [A&T]MLN) exhibited prolonged systemic persistence, remaining detectable for up to 24 h with comparable biodistribution profiles. Ex vivo fluorescence quantification of major organs at 4 h post‐injection further detailed the distribution patterns (Figure 7B,C). Free Cy5‐siRNA showed minimal organ retention, whereas LN accumulated intensely in the liver and kidneys, whereas AMLN, TMLN, and [A&T]MLN displayed broader but weaker distribution, with hepatic and renal fluorescence intensities reduced by approximately 50% compared to7 LN.

### 2.8 Biocompatibility and In Vivo Safety Profile

The cytotoxicity of [A&T]MLN was evaluated in HEK293T‐ACE2 and THP‐1 cells using a CCK‐8 assay. After 24 h of incubation, cell viability exceeded 90% across the tested concentration range of siRNA (150–750 nm) in both cell lines, without any formulation inducing a significant reduction when compared with the untreated control (Figure S3).

**FIGURE 8:**
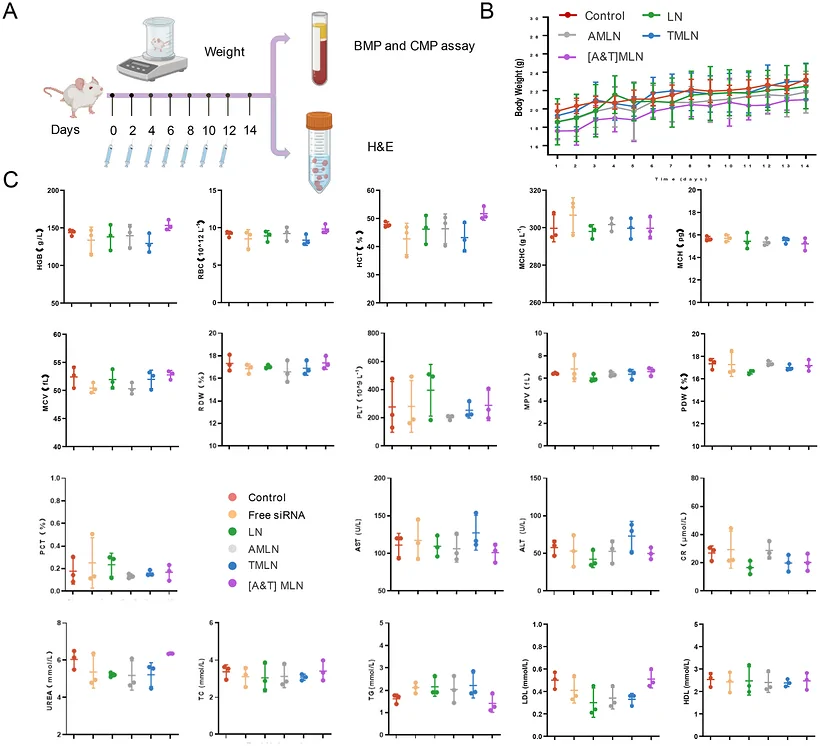
In vivo safety assessment of [A&T]MLN. (A) Schematic illustration of the repeated‐dose safety study timeline in PBMC‐humanized mice. BMP: blood metabolic panel; CMP: comprehensive metabolic panel. (B) Body weight changes following intravenous administration of PBS or siRNA‐loaded nanoparticles (every 48 h; siRNA dose: 1.2 mg/kg). Data are presented as mean ± SD (*n* = 3 per group). Statistical significance was assessed by one‐way ANOVA. No significant differences were found between any treatment group and the control group (all *p* > 0.05). (C) Serum biochemical analysis on day 14. Data are presented as mean ± SD (*n* = 3 per group). Statistical significance was assessed by one‐way ANOVA. No significant differences were found between any treatment group and the control group (all *p* > 0.05).

**FIGURE 9:**
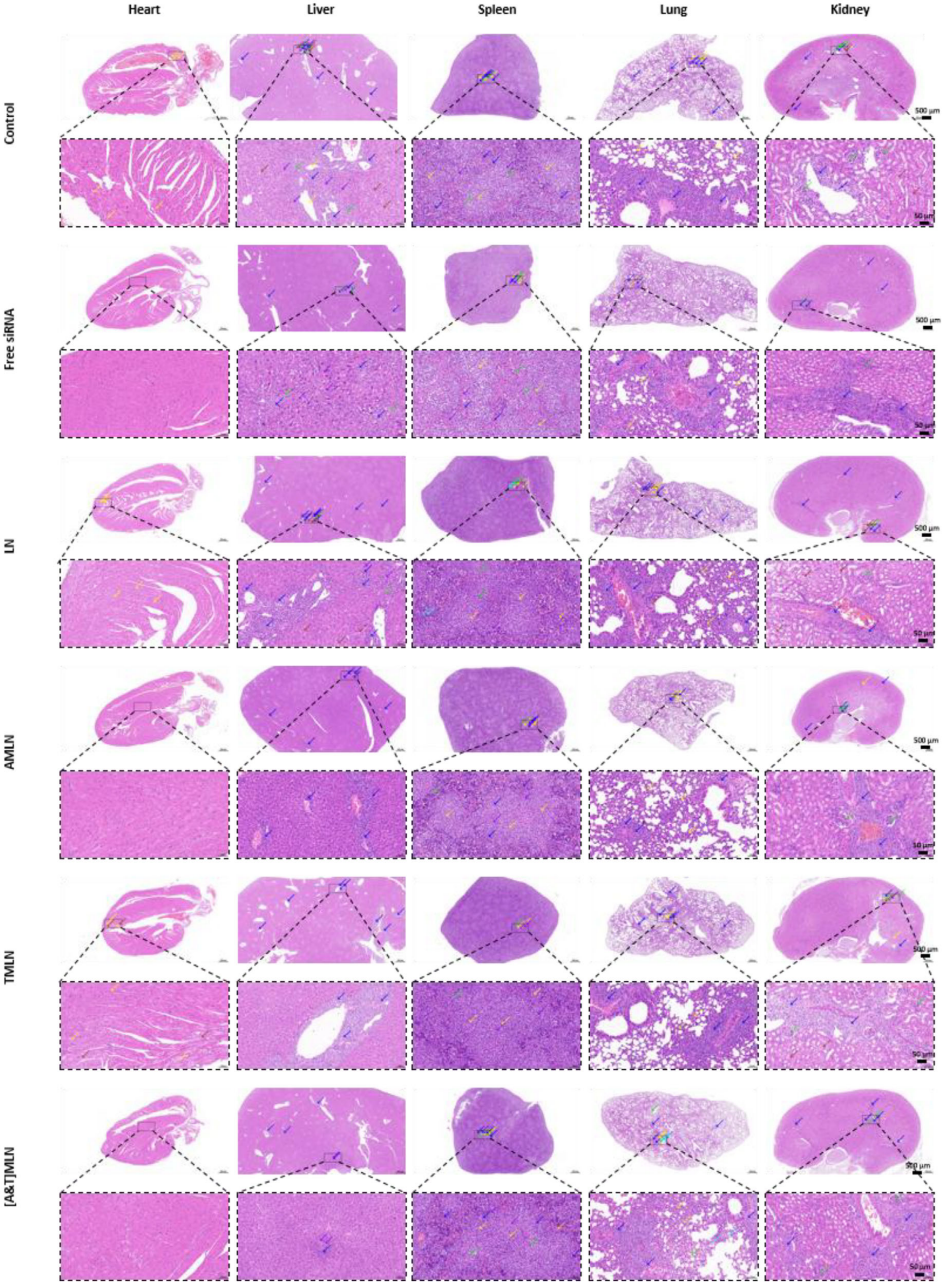
Histopathological evaluation of major organs in the in vivo safety assessment. Representative hematoxylin and eosin (H&E)‐stained sections of the heart, liver, spleen, lung, and kidney from PBMC‐humanized mice treated with PBS, free siRNA, LN, AMLN, TMLN, or [A&T]MLN. For each organ, whole‐section (or low‐magnification) images and corresponding high‐magnification fields are shown. Black boxes and dashed lines indicate the regions magnified in the adjacent high‐power views. Colored arrows denote representative histopathological features, with organ‐specific annotations as follows: in the heart, yellow and brown arrows indicate cardiomyocyte vacuolar degeneration and edema, respectively; in the liver, brown arrows indicate hepatocellular edema, purple arrows indicate punctate necrosis, blue arrows indicate lymphocytic/granulocytic cuffing or focal inflammatory infiltration, yellow arrows indicate bile duct proliferation, and green arrows indicate connective tissue proliferation; in the spleen, yellow arrows indicate macrophage proliferation/replacement, purple arrows indicate necrotic debris, green arrows indicate connective tissue proliferation, blue arrows indicate granulocytic infiltration, and cyan arrows indicate extramedullary hematopoiesis; in the lung, yellow arrows indicate granulocytic infiltration of alveolar walls, blue arrows indicate perivascular/peribronchiolar lymphocytic cuffing or focal infiltration, green arrows indicate focal alveolar hemorrhage, cyan arrows indicate macrophage exudation, purple arrows indicate necrotic cellular debris, and brown arrows indicate bronchiolar epithelial edema, epithelial desquamation, or goblet cell hyperplasia; and in the kidney, green arrows indicate mesangial matrix expansion or mesangial cell proliferation, brown arrows indicate tubular epithelial edema, yellow arrows indicate tubular dilation, and blue arrows indicate perivascular lymphocytic infiltration. Scale bars: low‐magnification overviews, 100 µm; high‐magnification insets, 50 µm.

To evaluate the long‐term in vivo safety of [A&T]MLN, PBMC‐humanized mice received repeated administrations of PBS, free siRNA, LN, AMLN, TMLN, or [A&T]MLN. Longitudinal assessments were performed, including body weight, hematological parameters, serum biochemistry, and histopathological evaluation of major organs (Figures 8A–C, 9 and Table S1). No treatment‐related alterations in body weight, complete blood counts, or serum biochemical indices were observed across any of the groups examined.

Representative hematoxylin and eosin (H&E)‐stained whole‐section and high‐magnification images of the heart, liver, spleen, lung, and kidney from each group are shown in Figure 9. In the [A&T]MLN group, pulmonary histology revealed scattered granulocytic infiltration along the alveolar walls, mild widening and thickening of the alveolar septa, perivascular and peribronchiolar lymphocytic cuffing, focal alveolar hemorrhage, and limited macrophage exudation. Hepatic and splenic alterations in this group included lymphocytic cuffing or focal lymphocytic infiltration, mild hepatocellular edema with occasional punctate necrosis, disruption of red and white pulp architecture, lymphocyte depletion, macrophage replacement, necrotic debris, and connective tissue proliferation. Notably, all these histopathological changes fell within the spectrum of model‐associated lesions observed across treatment groups. Cardiac sections from the [A&T]MLN group showed preserved endocardial, myocardial, and epicardial architecture without overt abnormalities, whereas kidney sections exhibited evenly distributed glomeruli, mild mesangial proliferation, largely preserved tubular epithelial morphology, and perivascular lymphocytic infiltration.

To enable quantitative comparison between groups of pathological changes, histopathological alterations in the organs showing the most pronounced changes, namely, the lung, liver, and spleen, were assessed in a blinded manner using a validated semi‐quantitative GVHD scoring system (Table S2) [21]. The lung, liver, and spleen scores in the [A&T]MLN group were 3.5, 3.5, and 3.5, respectively, identical to those recorded for the control group. These findings indicate that the observed organ abnormalities in the [A&T]MLN group were neither associated with increased GVHD severity relative to controls, nor reflective of a formulation‐specific toxicity profile.

Collectively, the in vitro cytotoxicity data and the in vivo histopathological assessments consistently demonstrate that [A&T]MLN did not impair cell viability at the tested concentrations (150–750 nm) and, upon repeated administration, neither exacerbated the underlying xGVHD‐like pathology in PBMC‐humanized mice nor induced detectable systemic toxicity at the dose evaluated.

## 3 Discussion

The development of therapeutics that concurrently address viral replication and dysregulated host inflammation remains a major challenge in treating severe COVID‐19 [22, 23, 24]. To address this need, we developed a dual‐membrane biomimetic nanoplatform ([A&T]MLN) designed for triple‐modal action: broad‐spectrum viral neutralization, targeted anti‐inflammatory activity, and intracellular delivery of therapeutic siRNA.

Our design rationale was grounded in the key pathological drivers of SARS‐CoV‐2 infection [25, 26]. The selection of membranes from ACE2‐overexpressing cells provided a high‐density array of viral entry receptors, intended to function as decoys. This was confirmed by the nanomolar affinity of [A&T]MLN for the viral spike protein and its potent, dose‐dependent neutralization of both pseudotyped and authentic variants. Notably, this neutralization achieved 100% breadth across a panel of six pseudotyped and three authentic variants, highlighting the strategic advantage of targeting the conserved ACE2‐binding mechanism rather than mutable viral epitopes [27, 28, 29]. We acknowledge, however, that more recently emerged lineages such as XBB, EG.5, and JN.1 were not included in our test panel. SARS‐CoV‐2 continues to evolve, and some of these lineages exhibit substantial immune escape and altered ACE2 binding affinity. Nevertheless, extensive structural and biochemical evidence indicates that all these lineages retain the ability to bind human ACE2 for host cell entry. For example, XBB sub‐lineages (e.g., XBB.1.5, XBB.1.16) maintain ACE2 binding, albeit with potentially reduced affinity. BA.2.86 displays strong ACE2 binding, whereas its JN.1 descendant exhibits reduced but still measurable ACE2 interaction. EG.5, which derives from the XBB branch, also retains ACE2 binding [30, 31]. Given that [A&T]MLN functions as an ACE2‐displaying nanodecoy rather than targeting specific spike epitopes, we anticipate that the platform will retain neutralizing activity against any ACE2‐dependent variant, although the potency (e.g., IC_50_ values) may vary depending on the variant's intrinsic ACE2 binding affinity.

Concurrently, to counteract the immunopathology of severe disease, we incorporated THP‐1‐derived macrophage membranes. This endowed the nanoplatform with a diverse repertoire of cytokine receptors (e.g., IL‐6R and TNF‐R1), enabling efficient and dose‐dependent scavenging of key inflammatory mediators such as IL‐6 and TNF‐α, in vitro [32, 33, 34]. Notably, while [A&T]MLN exhibited high neutralization efficiency against primary pyrogenic cytokines including IL‐6 (76.2%), IL‐1β (82.1%), and TNF‐α (77.7%), its capacity to neutralize GM‐CSF was considerably lower (19.6%). This marked discrepancy is likely attributable to two main factors. First, the relatively low native expression level of CD116 on THP‐1 cells, which is approximately ten‐fold lower than that of IL6R according to The Human Protein Atlas; Second, inherent differences in receptor‐ligand binding stoichiometry between GM‐CSF and its receptor complex compared with the other cytokine‐receptor pairs. Together, these observations highlight a fundamental limitation of cell‐membrane‐coated nanoplatforms: their cytokine‐scavenging profile is inherently constrained by the donor cell's native proteome.

The in vivo anti‐inflammatory efficacy of [A&T]MLN was substantial. In a murine model of SARS‐CoV‐2 spike protein‐induced acute lung injury, which recapitulates key features of COVID‐19‐associated immunopathology [35], treatment with [A&T]MLN initiated at 36 h post‐model construction significantly attenuated disease severity. This 36‐h time point was deliberately selected to target the critical “hyper‐acute therapeutic tipping point,” a window in which viral replication is actively accelerating toward its peak and the host immune response is priming the cytokine cascade, yet pulmonary tissue still retains sufficient structural plasticity for effective intervention. The therapeutic benefits of [A&T]MLN were evidenced by the reversal of weight loss, reduction in pulmonary inflammatory infiltrates and cytokine levels, and near‐complete restoration of lung architecture, as assessed by histopathological scoring. Moreover, the superior protection afforded by [A&T]MLN and TMLN, compared with AMLN, directly highlights the indispensable role of the macrophage membrane component in mitigating cytokine storm‐driven tissue injury.

Although TMLN (macrophage membrane alone) performs comparably to [A&T]MLN in anti‐inflammatory evaluation both in vitro and in vivo, relying solely on TMLN is fundamentally insufficient for comprehensive COVID‐19 therapy in real‐world clinical settings, where the pathophysiology is driven by two parallel but interconnected processes: uncontrolled viral replication and dysregulated host hyperinflammation. In this context, viral entry and intracellular replication serve as the upstream “trigger” that continuously fuels the downstream cytokine storm “outcome” [36, 37]. Although TMLN can transiently scavenge existing cytokines, it cannot prevent ongoing viral entry and replication. Consequently, viruses multiplying within host cells will inevitably provoke subsequent, often more severe waves of de novo cytokine release, rendering TMLN alone incapable to break the self‐perpetuating cycle of immunopathology. By integrating the ACE2 membrane with the macrophage membrane, [A&T]MLN simultaneously quenches the upstream viral trigger and attenuates the downstream inflammatory cascade. This dual‐action design effectively disrupts the vicious cycle of virus‐driven hyperinflammation, an advantage that neither AMLN (ACE2 membrane alone) nor TMLN can achieve independently. Importantly, the incorporation of the ACE2 membrane does not compromise the potent anti‐inflammatory activity conferred by the macrophage membrane. Instead, it adds the indispensable antiviral arm that TMLN entirely lacks. Thus, the dual‐membrane design is essential for achieving a truly comprehensive, triple‐modal therapy that addresses both arms of COVID‐19 pathophysiology.

The successful fabrication of a functional hybrid membrane was a critical step in our design. Colocalization imaging and FRET analysis confirmed the genuine fusion of the two source membranes, rather than a simple mixture [38, 39]. This engineered membrane was subsequently coated onto siRNA‐loaded lipid nanoparticles to generate the final [A&T]MLN construct. Comprehensive characterization verified the preservation of key protein structure and function post‐fabrication, while also revealing desirable pharmaceutical properties: excellent colloidal stability, sustained siRNA release profile (> 80% within 24 h), and a net positive surface charge conducive to cellular interaction [40, 41]. Notably, the cumulative siRNA release from the membrane‐decorated LN ([A&T]MLN) was significantly higher than that from bare LN. This difference is not attributable to differences in loading capacity, as bare LN exhibited high encapsulation efficiency (99.9 ± 0.9%). Two mechanisms account for the enhanced siRNA release from the membrane‐coated LN. First, mechanical force‐induced membrane rearrangement and structural relaxation occur during the co‐extrusion process, in which fused cell membrane fragments and pre‐formed bare LN cores are co‐extruded through polycarbonate membranes. This process generates metastable hybrid interfaces and nano‐scale defects on the surface shell, which, upon exposure to the release medium, facilitate faster siRNA diffusion compared with the rigidly packed bare LN. Second, the incorporation of negatively charged cell membranes weakens the electrostatic interactions between siRNA and the cationic lipids within the LN core, thereby promoting more efficient siRNA release from the membrane‐decorated LN under physiological conditions.

A significant hurdle for systemic nanotherapeutics is rapid clearance by the mononuclear phagocyte system. [A&T]MLN was engineered to overcome this through biomimetic “self‐marking”, attributed in part to the presence of CD47 on the macrophage membrane [42, 43]. This design proved successful in vitro, where [A&T]MLN exhibited significantly reduced uptake by macrophages compared with uncoated nanoparticles. While the in vitro uptake studies were performed exclusively using THP‐1‐derived macrophages, the CD47‐mediated immune evasion mechanism is expected to broadly suppress phagocytosis by other SIRPα‐expressing cells, including liver‐resident Kupffer cells [44, 45]. This is further supported by the significantly reduced hepatic accumulation of membrane‐coated nanoparticles observed in vivo, with [A&T]MLN achieving prolonged circulation (detectable for up to 24 h) and reduced non‐specific accumulation in the liver and kidneys of PBMC‐humanized mice—a critical feature for enhancing therapeutic delivery to sites of infection and inflammation [46, 47, 48]. Direct evidence of inflammation‐driven lung accumulation is supported by the robust therapeutic efficacy observed in the ALI model (Figure 5), where [A&T]MLN significantly reduced BALF cytokines, protein leakage, and lung injury scores. These functional outcomes, together with published reports [49, 50] demonstrating the intrinsic chemotactic properties of macrophage membrane‐coated nanoparticles, suggest that [A&T]MLN exhibits active tropism toward inflamed lung tissue. Given that [A&T]MLN is intentionally engineered without any conventional, anatomically driven lung‐targeting ligands, it is reasonable that no quantitative spike or active accumulation in the lungs of healthy mice was observed. Rather than indiscriminately accumulating in healthy organs, the core therapeutic value of [A&T]MLN lies in its ability to accurately sense, home in on, and lock onto the specific microenvironment disrupted and infected by SARS‐CoV‐2. This highly precise pathological tropism is collectively driven by the dual‐functional components of the hybrid membrane shell, which naturally inherits critical inflammatory cytokine receptors from the THP‐1‐derived macrophage membrane and overexpressed ACE2 from the HEK‐293T‐ACE2 cell membrane. This disease‐activated accumulation in the lung is robustly validated by the therapeutic outcomes in the SARS‐CoV‐2 spike protein‐induced ALI mouse model. Therefore, [A&T]MLN is expected to accumulate at infected lesions rather than in healthy tissues, enabling precise delivery of its therapeutic cargo.

Finally, we confirmed the functional intracellular delivery of the third therapeutic modality: siRNA. Live‐cell confocal tracking demonstrated the classic trajectory of endocytic uptake, endolysosomal entrapment, and subsequent escape into the cytosol by 12 h post‐treatment. This efficient cytosolic release, facilitated by the proton‐sponge effect of the cationic lipid core, establishes the prerequisite for the siRNA payload to engage the RNAi machinery. Beyond physical delivery, the siRNA used in this study (C6G25S) is specifically designed to target a highly conserved region of the RNA‐dependent RNA polymerase (RdRp) of SARS‐CoV‐1/2. The siRNA target site of C6G25S remains highly conserved across the representative strains tested, including Wuhan‐Hu WT, B.1.617.2, BA.5, BA.1, XBB.1.9.1, and BA.2.86.1. Its potent antiviral activity has been previously validated: C6G25S covers 99.8% of SARS‐CoV‐2 variants and inhibits dominant strains (including Alpha, Delta, Gamma, and Epsilon) at picomolar IC_50_ ranges in vitro [51]. These attributes, combined with the confirmed cytosolic release, position [A&T]MLN to potently suppress intracellular viral replication. Thus, [A&T]MLN offers a delayed but complementary wave of intracellular antiviral defense alongside immediate extracellular neutralization [52, 53].

Although certain histopathological alterations were observed, the nanoplatform was considered to be safe, as no significant differences in cytotoxicity or pathological changes were observed compared with those in the control group. However, an important consideration when interpreting the safety profile of [A&T]MLN is the intrinsic biological limitation of the PBMC‐humanized mouse model. Although PBMC‐humanized immunodeficient mice enable the evaluation of human immune cell‐mediated responses in vivo, they are inherently susceptible to xenogeneic graft‐versus‐host disease (xGVHD)‐like pathology, wherein transplanted human T cells may recognize murine tissues and infiltrate multiple organs, including the lung, liver, spleen, kidney, and heart [54, 55]. This model‐associated pathology is reflected in the representative histological images presented in Figure 9, where abnormalities were not restricted to the [A&T]MLN group but were observed across multiple treatment groups, particularly in the lung, liver, and spleen, which are recognized target organs of xGVHD. Accordingly, terminal histopathological findings in PBMC‐humanized mice should not be interpreted in isolation as direct evidence of nanoparticle‐induced toxicity; rather, they must be evaluated in the context of model‐associated background pathology, relevant formulation controls, and blinded semi‐quantitative xGVHD scoring. Previous clinicopathological studies have identified the lung, liver, and spleen as major target organs for xGVHD assessment and have demonstrated that histopathological scores correlate with clinical severity and the extent of human T cell engraftment within tissues, with mild baseline histological abnormalities reported even in untreated or control animals [21, 56]. In the present study, the xGVHD scores for the lung, liver, and spleen in the [A&T]MLN group were not higher than those in the PBS control group, and descriptive histopathological examination of the heart and kidney revealed no formulation‐specific structural injury. Together with the absence of treatment‐related changes in body weight, hematological parameters, and serum biochemical indices, these findings collectively indicate that repeated administration of [A&T]MLN did not exacerbate the background xGVHD‐like pathology of PBMC‐humanized mice nor induce detectable treatment‐related systemic toxicity under the tested dosing regimen.

In conclusion, we have developed a biomimetic nanoplatform that co‐engages three complementary therapeutic actions against SARS‐CoV‐2 infection. By functionally integrating viral decoy receptors, cytokine‐scavenging membranes, and a protected siRNA payload into a single entity with immune‐evasive properties, [A&T]MLN simultaneously targets viral entry, intracellular replication, and dysregulated host inflammation. This multi‐pronged strategy addresses the core interconnected pathologies of severe COVID‐19 and provides a versatile and robust platform that may be adaptable to combating other viral infections characterized by hyperinflammation. Future work will focus on evaluating the combined therapeutic efficacy of all three modalities in advanced infection models and exploring the platform's potential against other emerging pathogens.

## 4 Method

### 4.1 Materials

1,2‐Dioleoyl‐sn‐glycero‐3‐phosphocholine (DOPC) and 1,2‐di‐O‐octadecenyl‐3‐trimethylammonium propane (DOTMA) were purchased from Shanghai Advanced Vehicle Technology Pharmaceutical Ltd. (Shanghai, China). siRNA C6G25S (sense: 5′‐mCsmUsmGmUfCmAfAfAfCfCmCmGmGmUmAmAmUsmUsmU‐3′; antisense: 5′‐mAsfAsmAfUfUmAmCfCfGmGmGmUmUfUmGfAmCfAmGsmUsmU‐3′), negative control siRNA (sense: 5′‐UUCUUCGAACGUGUCACGUTT‐3′; antisense: 5′‐ACGUGACACGUUCGGAGAATT‐3′), and Cy5‐siRNA were obtained from GenePharma (Shanghai, China). RPMI 1640 medium and fetal bovine serum (FBS) were acquired from the Cell Resource Centre (IBMS, CAMS/PUMC, Beijing, China). Antibodies against ACE2, IL‐6R, TNF‐R1, IL‐1R, CD116, and CD47, along with horseradish peroxidase (HRP)‐conjugated goat anti‐rabbit IgG, were purchased from Abcam (Cambridge, UK). SARS‐CoV‐2 (2019‐nCoV) Spike S1+S2 ECD recombinant protein was sourced from Sino Biological Inc. (Beijing, China). DiI and Hoechst 33258 were obtained from Beyotime Biotech Inc. (Shanghai, China). Recombinant human IL‐6, IL‐1β, TNF‐α, and GM‐CSF were purchased from PeproTech (Rocky Hill, USA). THP‐1 and RAW264.7 cells were provided by the Cell Resource Centre (IBMS, CAMS/PUMC, Beijing, China). The HEK‐293T‐ACE2 cell line was acquired from Yisheng Biotechnology Shanghai Co., Ltd. (Shanghai, China). Recombinant S1SP was purchased from RayBiotech (Atlanta, USA). Pseudoviruses encoding the spike proteins of the WT, Alpha (B.1.1.7), Beta (B.1.351), Gamma (P.1), Delta (B.1.617.2), and Omicron (BA.4/5) variants were all obtained from Zhongyan Guobang Technology Co., Ltd. (Beijing, China). The SARS‐CoV‐2 WT strain (IME‐BJ01 strain, GenBank No. MT291831), its Beta variant (CSTR: 16698.06.NPRC2.062100001), and the Omicron BA.5 variant (SARS‐CoV‐2 strain Omicron CoV/human/CHN_CVRI‐12/2022) were obtained from the Chinese Academy of Agricultural Sciences (Changchun, China). Red blood cell lysis buffer was sourced from Solaibao Technology Co., Ltd. (Beijing, China). All SARS‐CoV‐2 WT strains were handled in a biosafety level 3 (BSL‐3) laboratory. Virus stocks were propagated and titrated on Vero E6 cells.

Male PBMC‐humanized mice (4‐5 weeks old) were purchased from Shanghai Model Organisms Center, Inc. (Shanghai, China). Male K18‐hACE2‐2A‐CreERT2 mice (6‐8 weeks old) were obtained from Saiye Model Biological Research Center Co., Ltd. (Taicang, China). All animal experiments were conducted in accordance with the ethical guidelines approved by the Animal Care and Use Ethics Committee of the Academy of Military Medical Sciences (IACUC‐DWZX‐2022‐668). All animals were randomly assigned to experimental groups based on body weight.

### 4.2 Isolation of Cell Membranes

HEK293T‐ACE2 and native HEK293T cell membranes were isolated as described previously [57, 58]. Cells were harvested and subjected to three freeze‐thaw cycles. Membranes were pelleted by centrifugation (6000 × g, 10 min, 4°C), washed with ice‐cold PBS containing protease inhibitors, and sonicated on ice (120 W, 10 min). The membrane fraction was subsequently collected by ultracentrifugation (100 000 × g, 10 min), washed, and stored at −80°C.

THP‐1 cell membranes were prepared with minor modifications to the previously reported protocol [59]. Cells were homogenized with a Dounce homogenizer (20 passes). Lysates were centrifuged (800 × g, 5 min), and the supernatant was further centrifuged (10 000 × g, 25 min). The resulting supernatant was ultracentrifuged (150 000 × g, 50 min) to pellet plasma membranes, which were washed and stored at −80°C.

Membrane protein concentration was determined using a bicinchoninic acid (BCA) assay.

### 4.3 Membrane Fusion Validation

Membrane fusion was validated by CLSM and FRET [60, 61]. For CLSM, HEK293T‐ACE2 and THP‐1 membranes were separately labeled with DiI (red) and DiO (green), respectively. Labeled membranes were mixed and sonicated (150 W, 5 min) to induce fusion, with a non‐sonicated mixture serving as the control. Samples were air‐dried on glass slides and imaged by CLSM.

For FRET analysis, THP‐1 membranes were dual‐labeled with DiI (donor) and DiD (acceptor) [60, 61]. Labeled membranes were then mixed with HEK293T‐ACE2 membranes at varying protein weight ratios (5:1, 3:1, 1:1, and 0:1), followed by sonication (150 W, 5 min). Fluorescence spectra were recorded on a microplate reader with excitation at 520 nm and emission monitored over 550–750 nm. The fluorescence recovery of the donor dye (DiI on THP‐1 membranes) was used to confirm membrane fusion.

### 4.4 Preparation and Characterization of [A&T]MLN

Bare LN were prepared using the thin‐film hydration method [62, 63]. Briefly, the lipids DOPC and DOTMA (Shanghai Advanced Vehicle Technology Pharmaceutical Ltd., China) were mixed with cholesterol at a weight ratio of 85:10:5 in chloroform. After rotary evaporation to form a thin film, hydration was performed at 55°C for 45 min using HEPES buffer (20 mm HEPES, 150 mm NaCl, pH 7.0) containing a complex of protamine and target siRNA (siRNA C6G25S or Cy5‐siRNA; GenePharma, China) at a protamine:siRNA weight ratio of 1.5:1. The suspension was sonicated (45 W, 60 s), and free siRNA was removed by dialysis.

To prepare the hybrid membrane‐coated nanoplatform ([A&T]MLN), HEK293T‐ACE2 and THP‐1 cell membranes were mixed at a 1:1 protein weight ratio, fused by sonication (150 W, 5 min, ice bath), and coated onto pre‐formed LN by extrusion through 400‐nm and 200‐nm polycarbonate membranes using a liposome extruder (AVESTIN, Canada). AMLN and TMLN were prepared using the same procedure.

The morphology of the nanoparticles was examined by TEM (Hitachi HT7800, Japan) after negative staining with phosphotungstic acid. Hydrodynamic diameter and zeta potential were measured at 25°C using an Anton Paar Litesizer 500 (Austria).

### 4.5 siRNA Encapsulation and Release

The siRNA encapsulation efficiency was determined fluorometrically (λex/λem = 649/680 nm). Total siRNA was measured after lysing the nanoparticles with 10% (w/v) Triton X‐100. Free siRNA was separated using an ultrafiltration tube (50 kDa MWCO; Solaibao Technology Co., Ltd., China) and quantified. The siRNA encapsulation efficiency was calculated according to Equation (1):

(1) $$\mathrm{Encapsulation}\;\mathrm{efficiency}\;\;\%=\frac{W_{total}-W_{free}}{W_{total}}\;\times 100\%\;$$

where W_total_ represents the total amount of siRNA in the formulation, and W_free_ denotes the amount of free siRNA outside the nanoparticles.

In vitro siRNA release was studied using a dialysis method [62, 63]. Nanoparticle samples (0.5 mL) were loaded into dialysis bags (50 kDa MWCO) and immersed in 35 mL of PBS (pH 7.4) at 37°C under gentle shaking. At predetermined intervals, 0.7 mL of the external medium was collected and replaced with an equal volume of fresh PBS. The concentration of released Cy5‐siRNA in the collected aliquots was quantified by fluorescence spectrometry (λex = 640 nm, λem = 680 nm). The cumulative release percentage was calculated according to Equation (2):

(2) $$\mathrm{Cumulative}\;\mathrm{release}\;(\%)=\frac{\left(V\times C_{t}+V_{r}\times \sum C_{m}\right)}{Dose}\times 100\%$$

where V is the total volume of the release medium (35 mL), C_t_ is the measured siRNA concentration at time t, ΣC_m_ is the sum of siRNA concentrations in all previously collected samples, V_r_ is the volume removed at each sampling (0.7 mL), and Dose is the total amount of siRNA initially loaded into the dialysis bag.

### 4.6 Colloidal Stability

The colloidal stability of [A&T]MLN was evaluated in water, PBS (pH 7.4), and complete culture medium (RPMI 1640 supplemented with 10% FBS; acquired from the Cell Resource Centre) using a Turbiscan Lab Expert at 37°C over 72 h.

### 4.7 Protein Profiling

Membrane protein profiles were analyzed by SDS‐PAGE followed by Coomassie Blue staining or WB [58]. Proteins were extracted using a commercial total protein extraction kit and separated on 4%–12% Bis‐Tris gels using a Bio‐Rad electrophoresis system. Electrophoresis was performed at 80 V for 20 min, followed by 120 V for 1 h. For WB, proteins were transferred to a polyvinylidene difluoride membrane, incubated with primary antibodies against ACE2, IL‐6R, TNF receptor I, IL‐1R, CD116, and CD47 (all from Abcam, Cambridge, UK), and then probed with an HRP‐conjugated goat anti‐rabbit IgG secondary antibody (Abcam). Signals were detected using a ChemiDoc MP imaging system (Bio‐Rad, Hercules, CA, USA).

Protein secondary structure was analyzed by far‐UV CD spectroscopy on a Chirascan‐Plus spectrometer equipped with a 0.1 cm quartz cell. Spectra were recorded from 190 to 310 nm with a bandwidth of 5 nm. Each sample was measured at a protein concentration of approximately 0.3 mg/mL, and the final spectra represent the average of three consecutive scans.

### 4.8 Virus Binding Assays

The binding affinity between [A&T]MLN and SARS‐CoV‐2 spike protein was quantified by BLI on a ForteBio Octet Red 96 system (Sartorius BioAnalytical Instruments, Bohemia, NY, USA) [57]. Biotinylated SARS‐CoV‐2 (2019‐nCoV) Spike S1+S2 ECD protein (Sino Biological Inc., China) was immobilized on streptavidin biosensors. Serially diluted [A&T]MLN (62.5–1000 nm in PBS) was then applied, and the association and dissociation phases were monitored. Sensorgram data were fitted to a 1:1 Langmuir binding model to determine the K_D_.

For authentic virus binding, [A&T]MLN was incubated with SARS‐CoV‐2 wild‐type (WT, IME‐BJ01) or Omicron BA.5 variant (both from the Chinese Academy of Agricultural Sciences, China) for 2 h at 37°C [57]. Complex formation was assessed by changes in hydrodynamic diameter and zeta potential and further visualized by TEM.

### 4.9 In Vitro Cytokine Neutralization Assay

The cytokine‐neutralizing capacity of the nanoparticles was evaluated as described previously [64]. Recombinant human IL‐6, IL‐1β, TNF‐α, and GM‐CSF (all from PeproTech, USA) were incubated with [A&T]MLN, AMLN, or TMLN (0–10 mg/mL) in PBS for 30 min at 37°C. After pelleting the nanoparticles (15 000 × g, 15 min), residual cytokines in the supernatant were quantified using a commercial ELISA kit (Cloud‐Clone, China).

### 4.10 Virus Neutralization Assay

Pseudovirus neutralization was measured using a luciferase‐reporter assay [57]. Briefly, serially diluted [A&T]MLN or other formulations were incubated with pseudovirus (650 TCID_50_; Zhongyan Guobang Technology Co., Ltd., China) for 1 h at 37°C. The mixture was then transferred to HEK293T‐ACE2 cells seeded in 96‐well plates. After 24 h, luciferase activity was quantified using a dual‐luciferase assay system (Promega). Virus‐only and medium‐only wells served as positive and negative controls, respectively; all tests were performed in triplicate. This assay was used to determine the half‐maximal inhibitory concentration (IC_50_) of [A&T]MLN against a panel of pseudoviruses (WT, Alpha, Beta, Gamma, Delta, and Omicron BA.4/5) and to compare the relative neutralization potency of free siRNA, AMLN, TMLN, and [A&T]MLN against WT and Omicron BA.4/5 specifically.

Neutralization of authentic SARS‐CoV‐2 by [A&T]MLN was assessed by a viral‐titer reduction assay [65]. Vero E6 cells were pretreated with [A&T]MLN (19.5–78 µg/mL) for 2 h and then infected with the WT, Beta, or Omicron BA.5 strains at an MOI of 0.01. Culture supernatants were harvested 48 h post‐infection, serially diluted, and titrated on fresh Vero E6 monolayers in 96‐well plates. After 4 days, viral titers were calculated as the 50% tissue culture infectious dose (TCID_50_) using the Reed–Muench method.

### 4.11 In Vivo Acute Lung Injury Model and Anti‐Inflammatory Efficacy

An acute lung injury (ALI) mouse model mimicking COVID‐19 was established as described previously, with minor modifications [35]. Male K18‐hACE2‐2A‐CreERT2 mice (6–8 weeks old; Saiye Model Biological Research Center Co., Ltd., China) were anesthetized and administered 50 µL of SARS‐CoV‐2 S1SP (RayBiotech, USA) via intratracheal instillation at a dose of 400 mg/kg, followed by an air bolus (150 µL) to promote distribution. Mice were monitored during recovery and for 4 h post‐procedure.

Thirty‐six hours post‐induction, mice were randomly assigned to treatment groups (*n* = 3 per group). They received a single intravenous injection of [A&T]MLN, AMLN, TMLN, or LN (equivalent siRNA dose), while control mice received PBS. Body weight was monitored every 12 h for 72 h. At 36 h post‐treatment, mice were euthanized. BALF was collected from the left lung. Total protein concentration in the BALF supernatant was determined by BCA assay. Inflammatory cytokines (IL‐6, IL‐1β, and TNF‐α) were quantified using an Ella automated immunoassay system (ProteinSimple, USA). BALF cells were lysed with red blood cell lysis buffer, washed, resuspended in PBS, and counted with a hemocytometer.

The right lung was fixed in 10% paraformaldehyde, paraffin‐embedded, sectioned (5 µm), and stained with H&E. For each sample, three random fields were imaged at 20× magnification. Lung injury was semi‐quantitatively scored according to an established method [66]. Five histopathological parameters were evaluated in a blinded manner: (i) neutrophils in alveolar spaces, (ii) neutrophils in interstitial spaces, (iii) hyaline membranes, (iv) proteinaceous debris in airspaces, and (v) alveolar septal thickening. The weighted Lung Injury Score (LIS) was calculated as Equation (3):

(3) $$\begin{array}{ccc} \mathrm{LIS}\; & = & 20\times \left(i\right)+14\times \left(\mathrm{ii}\right)+7\times \left(\mathrm{iii}\right)+7\times \left(\mathrm{iv}\right)+2\times \left(v\right)\\ & & /\left(\mathrm{number}\;\mathrm{of}\;\mathrm{fields}\times 100\right) \end{array}$$

Scores range from 0 (no injury) to 1 (maximal lung injury).

### 4.12 In Vitro Cellular Uptake and Trafficking

Cellular uptake was evaluated in THP‐1‐derived macrophages as described previously [62]. THP‐1 cells were differentiated with 100 ng/mL PMA for 48 h and then incubated for 8 h with Cy5‐siRNA‐loaded formulations (300 nm siRNA) at 37°C under 5% CO_2_. For qualitative analysis, cells were fixed with 4% paraformaldehyde, mounted with 50% glycerol, and imaged by CLSM (Zeiss LSM 880). For quantitative analysis, cells were detached, washed, resuspended in cold PBS, and analyzed by FCM (BD FACSAria II).

Intracellular trafficking of siRNA delivered by [A&T]MLN was tracked over time by CLSM [62]. RAW264.7 cells were seeded in confocal dishes at 1.8 × 10^5^ cells per well. Following overnight culture, cells were treated with Cy5‐siRNA‐loaded [A&T]MLN (final siRNA concentration 300 nm) for 4, 6, 8, or 12 h. Cells were then washed with PBS and stained with LysoTracker Red for 30 min to label lysosomes. After fixation with 4% paraformaldehyde (20 min) and nuclear counterstaining with Hoechst 33258 (10 min), siRNA localization was visualized by CLSM.

### 4.13 In Vivo Biodistribution

Biodistribution was assessed in male PBMC‐humanized mice (4–5 weeks old; Shanghai Model Organisms Center, Inc., China) [67, 68]. The PBMC‐humanized model was generated by intravenous injection of PBMCs. Briefly, 5 × 10^6^ human PBMCs (viability > 90%) suspended in 200 µL of sterile PBS were administered to NOD‐scid IL2rg^−^/^−^ (NSG) mice via tail vein injection. To validate the efficiency and quality of functional human immune reconstitution, the proportion of human CD45^+^ cells in peripheral blood was quantified by flow cytometry during the critical window at weeks 2 to 3 post‐engraftment. According to established benchmarks, a peripheral hCD45^+^ chimerism level of ≥ 25% served as the quality control standard for verifying successful reconstitution of a functional humanized immune background [69, 70]. Only mice meeting this criterion were included in subsequent studies. Mice received a single intravenous injection of PBS or Cy5‐siRNA‐loaded formulations (siRNA dose: 1.2 mg/kg). One cohort was imaged in vivo at designated time points using an IVIS Lumina II system (PerkinElmer) to monitor real‐time fluorescence distribution. The other cohort was euthanized 4 h post‐injection for ex vivo imaging of harvested organs (brain, heart, lungs, liver, spleen, and kidneys).

### 4.14 Safety Studies

Cytotoxicity was evaluated using a CCK‐8 assay [63]. HEK293T‐ACE2 and THP‐1 cells were seeded in 96‐well plates (6000 cells/well). After overnight culture, cells were treated with free siRNA, LN, AMLN, TMLN, or [A&T]MLN at siRNA concentrations ranging from 150 to 750 nm. After 24 h, CCK‐8 solution was added, incubated for 1 h, and absorbance was measured at 450 nm using a microplate reader (Tecan Spark, Switzerland). Cell viability was expressed relative to untreated controls.

The long‐term safety of [A&T]MLN was assessed in male PBMC‐humanized mice (4–5 weeks old). Mice were randomly divided into six groups (*n* = 3 per group) and received intravenous injections of PBS or Cy5‐siRNA‐loaded formulations (siRNA dose: 1.2 mg/kg) every 48 h for 14 days. Body weight was recorded every two days. On day 14, blood was collected for serum biochemistry and complete blood count analysis. Major organs (heart, lung, liver, spleen, and kidney) were harvested for histopathological evaluation following H&E staining. The clinical severity of xGVHD was assessed using a recommended scoring system (Table S2) [21, 71]. Briefly, lung and liver xenogeneic GVHD‐like lesions were semi‐quantitatively scored on a 0–4 scale with 0.5‐point increments based on perivascular/peribronchiolar inflammatory cuffing, parenchymal infiltration, structural disruption, and necrotic lesions. Spleen lesions were scored using the same scale based on lymphocyte depletion, necrotic/apoptotic cell burden, hemolysis, and architectural disruption. Heart and kidney sections were evaluated descriptively for structural abnormalities, degenerative changes, and inflammatory infiltration.

### 4.15 Statistical Analysis

All data are presented as mean ± SD from at least three independent experiments. Comparisons among multiple groups were performed using one‐way ANOVA, followed by Tukey's post‐hoc test for multiple comparisons. All analyses were conducted using GraphPad Prism (version 8.4.2). A *p*‐value of < 0.05 was considered statistically significant.

## Author Contributions

**Hui Li**: writing – original draft, investigation, validation, methodology, formal analysis, data curation. **Bohan Zhang**: investigation, methodology, formal analysis, validation, data curation. **Chao Shang**: investigation, validation, methodology, formal analysis, data curation. **Yi Li**: formal analysis, methodology, validation. **Te Zhao**: validation, methodology, formal analysis. **Shengya Liu**: writing – review and editing. **Dan Liu**: validation, formal analysis. **Junwei Che**: validation, formal analysis. **Cuiling Zhang**: validation, methodology. **Pinghui Wu**: formal analysis, methodology. **Wenjie Yang**: formal analysis, validation. **Junjie Ding**: validation, formal analysis. **Yuhua Ran**: writing – review and editing. **Yanan Zhai**: writing – review and editing. **Haihua Xiao**: writing – review and editing. **Jing Gao**: writing – review and editing, supervision, conceptualization. **Xiao Li**: conceptualization, writing – review and editing, supervision, resources. **Chunsheng Gao**: writing – review and editing, supervision, conceptualization. **Lin Li**: conceptualization, writing – review and editing, supervision, resources. **Zhiping Li**: conceptualization, writing – review and editing, supervision, project administration.

## Conflicts of Interest

The authors declare no conflicts of interest.

## Supporting information




**Supporting file**: advs77581‐sup‐0001‐SuppMat.docx.

## Data Availability

The data that support the findings of this study are available on request from the corresponding author. The data are not publicly available due to privacy or ethical restrictions.
