# Supplementary material for: A Dual‐Membrane Biomimetic Nanoplatform Enables Triple‐Modal Therapy Against SARS‐CoV‐2 Through Viral Decoy, Inflammation Neutralizing, and Intracellular RNAi
Source: Adv Sci (Weinh). 2026 Sep 6:e77581. Online ahead of print. doi: 10.1002/advs.77581 (PMC13547693; doi:10.1002/advs.77581)
Supplement: Supplementary file 1 [file ADVS-9999-e77581-s001.docx]

**A Dual-Membrane Biomimetic Nanoplatform Enables Triple-Modal Therapy Against SARS-CoV-2 Through Viral Decoy, Inflammation Neutralizing, and Intracellular RNAi**

Hui Li^1,2,3#^, Bohan Zhang^2,4#^, Chao Shang^5#^, Yi Li^1,2#^, Te Zhao^1,2^, Shengya Liu^6^, Dan Liu^1,2^, Junwei Che^1,2^, Cuiling Zhang^5^, Pinghui Wu^1,2^, Wenjie Yang^1,2^, Junjie Ding^1,2^, Yuhua Ran^1,2^, Yanan Zhai^1,2^, Haihua Xiao,^7^ Jing Gao^1,2*^, Xiao Li^5*^, Chunsheng Gao^1,2*^, Lin Li^2,4*^, Zhiping Li^1,2*^

^1^State Key Laboratory of National Security Specially Needed Medicines, Beijing 100039, China

^2^Academy of Military Medical Sciences, Beijing 100850, China

^3^Department of Pharmacy, Peking University Third Hospital, Beijing, 100191, China

^4^State Key Laboratory of Pathogen and Biosecurity, Beijing 100071, China

^5^Changchun Veterinary Research Institute, Chinese Academy of Agricultural Sciences, Changchun 130062, China

^6^Shenzhen International Travel Health Care Center (Shenzhen Customs District Port Outpatient Clinics), Shenzhen 518045, China

^7^Institute of Chemistry Chinese Academy of Sciences, Beijing 100190, China

^#^These four authors contribute equally.

^*^Corresponding authors: Zhiping Li, [dearwood2010@126.com;](mailto:dearwood2010@126.com;) Lin Li, [dearwood@sina.com.cn;](mailto:dearwood2010@126.com;) Chunsheng Gao, [largedna@163.com;](mailto:largedna@163.com;) Xiao Li, [skylee6226@163.com;](mailto:skylee6226@163.com;) Jing Gao, 13817029922@126.com.

**Fig. S1. Validation of membrane purity by confocal microscopy.** Representative CLSM images of membranes from **ACE2‑overexpressing HEK293T cells (A)**, parental **HEK293T cells (B)**, and **THP-1-derived macrophages (C)** before and after the extraction process. Nuclei were stained with Hoechst 33258 (blue). Cell membranes were labeled with the lipophilic dye DiI (red). Scale bar, 5 μm.

**Fig. S2. Batch-to-batch consistency of ACE2 expression in isolated HEK293T-ACE2 membranes. (A)** Western blot analysis of ACE2 protein in vesicle samples derived from HEK293T-ACE2 cells across three independent extraction batches. **(B)** ELISA quantification of ACE2 protein levels in the same three batches. Data are presented as mean ± SD (n = 3). Statistical significance was assessed by one‑way ANOVA, with no significant differences detected among the batches.

**Fig. S3. Cytotoxicity of nanoparticle formulations.** Cell viability of HEK293T‑ACE2 cells and THP‑1 macrophages after 24 h treatment with free siRNA or the indicated nanoparticle formulations (LN, AMLN, TMLN, and [A&T]MLN) at siRNA concentrations ranging from 150 to 750 nM. Data are presented as mean ± SD (n = 3 per group). Statistical significance was assessed by one‑way ANOVA followed by Tukey’s post-hoc test for multiple comparisons; no significant differences were observed between any treatment group and the untreated control.

**Table S1 Statistical analysis and multiple comparisons of *in vivo* safety parameters.**

| **Parameter** | **Control vs Free siRNA** | **Control vs LN** | **Control vs AMLN** | **Control vs TMLN** | **Control vs [A&T] MLN** |
| --- | --- | --- | --- | --- | --- |
| **HGB (g/L)** | 0.9312 | 0.9939 | 0.9988 | 0.7635 | 0.9397 |
| **RBC (10¹²/L)** | 0.9194 | 0.9987 | >0.9999 | 0.8213 | 0.9027 |
| **HCT (%)** | 0.7201 | 0.9970 | 0.9978 | 0.7839 | 0.8839 |
| **MCHC (g/L)** | 0.7296 | 0.9993 | 0.9983 | >0.9999 | >0.9999 |
| **MCH (pg)** | >0.9999 | 0.9828 | 0.9505 | 0.9962 | 0.7650 |
| **MCV (fL)** | 0.5184 | 0.9981 | 0.4700 | 0.9986 | 0.9994 |
| **RDW (%)** | 0.9349 | 0.9899 | 0.6681 | 0.9514 | >0.9999 |
| **PLT (10⁹/L)** | >0.9999 | 0.8906 | 0.9835 | >0.9999 | >0.9999 |
| **MPV (fL)** | 0.9334 | 0.9334 | >0.9999 | >0.9999 | 0.9986 |
| **PDW (%)** | >0.9999 | 0.5808 | >0.9999 | 0.9705 | 0.9987 |
| **PCT (%)** | 0.9653 | 0.9880 | 0.9964 | >0.9999 | >0.9999 |
| **AST (U/L)** | 0.9982 | >0.9999 | 0.9995 | 0.8875 | 0.9828 |
| **ALT (U/L)** | 0.9988 | 0.7857 | 0.9980 | 0.7968 | 0.9849 |
| **CR (μmol/L)** | 0.9984 | 0.5578 | 0.9996 | 0.8385 | 0.8704 |
| **UREA (mmol/L)** | 0.7189 | 0.5405 | 0.5016 | 0.5483 | 0.9846 |
| **TC (mmol/L)** | 0.9885 | 0.9717 | 0.9904 | 0.9814 | >0.9999 |
| **TG (mmol/L)** | 0.7278 | 0.6584 | 0.8426 | 0.5656 | 0.9891 |
| **LDL (mmol/L)** | 0.8461 | 0.1754 | 0.3641 | 0.3070 | >0.9999 |
| **HDL (mmol/L)** | 0.9994 | >0.9999 | 0.9987 | 0.9975 | >0.9999 |

*Note: All adjusted *P* values were determined using Tukey’s multiple comparisons test following one-way ANOVA. No significant differences were observed between the control group and any treatment group (all *P* > 0.05).

**Table S2. Blinded semi-quantitative histopathological scoring of xenogeneic GVHD-like lesions** in PBMC-humanized mice.

| **Group** | **Lung score** | **Liver score** | **Spleen score** |
| --- | --- | --- | --- |
| **Control** | 3.5 | 3.5 | 3.5 |
| **Free siRNA** | 4.0 | 4.0 | 4.0 |
| **LN** | 4.0 | 3.0 | 3.5 |
| **AMLN** | 3.0 | 3.5 | 3.0 |
| **TMLN** | 4.0 | 4.0 | 3.5 |
| **[A&T]MLN** | 3.5 | 3.5 | 3.5 |

Note: H&E-stained lung, liver, and spleen sections were independently evaluated using a semi-quantitative xenogeneic GVHD histopathological scoring system. Lung and liver sections were scored based on perivascular/peribronchiolar inflammatory cuffing, parenchymal infiltration, structural disruption, and necrotic lesions; spleen sections were scored based on lymphocyte depletion, necrotic/apoptotic cell burden, hemolysis, and architectural disruption.
